# A Novel Cationic Microbubble Coated with Stearic Acid-Modified Polyethylenimine to Enhance DNA Loading and Gene Delivery by Ultrasound

**DOI:** 10.1371/journal.pone.0076544

**Published:** 2013-09-26

**Authors:** Qiaofeng Jin, Zhiyong Wang, Fei Yan, Zhiting Deng, Fei Ni, Junru Wu, Robin Shandas, Xin Liu, Hairong Zheng

**Affiliations:** 1 Paul C. Lauterbur Research Center for Biomedical Imaging, Institute of Biomedical and Health Engineering, Shenzhen Institutes of Advanced Technology, Chinese Academy of Sciences, Shenzhen, China; 2 Department of Physics, University of Vermont, Burlington, Vermont, United States of America; 3 Department of Bioengineering, University of Colorado Denver Anschutz Medical Campus, Aurora, Colorado, United States of America; 4 Shenzhen Key Lab for MRI, Shenzhen Institutes of Advanced Technology, Chinese Academy of Sciences, Shenzhen, China; Case Western Reserve University, United States of America

## Abstract

A novel cationic microbubble (MB) for improvement of the DNA loading capacity and the ultrasound-mediated gene delivery efficiency has been developed; it has been prepared with commercial lipids and a stearic acid modified polyethylenimine 600 (Stearic-PEI600) polymer synthesized via acylation reaction of branched PEI600 and stearic acid mediated by N, N'-carbonyldiimidazole (CDI). The MBs’ concentration, size distribution, stability and zeta potential (ζ-potential) were measured and the DNA loading capacity was examined as a function of the amount of Stearic-PEI600. The gene transfection efficiency and cytotoxicity were also examined using breast cancer MCF-7 cells via the reporter plasmid pCMV-Luc, encoding the firefly luciferase gene. The results showed that the Stearic-PEI600 polymer caused a significant increase in magnitude of ζ-potential of MBs. The addition of DNA into cationic MBs can shift ζ-potentials from positive to negative values. The DNA loading capacity of the MBs grew linearly from (5±0.2) ×10^−3 ^pg/µm^2^ to (20±1.8) ×10^−3 ^pg/µm^2^ when Stearic-PEI600 was increased from 5 mol% to 30 mol%. Transfection of MCF-7 cells using 5% PEI600 MBs plus ultrasound exposure yielded 5.76±2.58×10^3 ^p/s/cm^2^/sr average radiance intensity, was 8.97- and 7.53-fold higher than those treated with plain MBs plus ultrasound (6.41±5.82) ×10^2^ p/s/cm^2^/sr, (P<0.01) and PEI600 MBs without ultrasound (7.65±6.18) ×10^2^ p/s/cm^2^/sr, (P<0.01), respectively. However, the PEI600 MBs showed slightly higher cytotoxicity than plain MBs. The cells treated with PEI600-MBs and plain MBs plus ultrasound showed 59.5±6.1% and 71.4±7.1% cell viability, respectively. In conclusion, our study demonstrated that the novel cationic MBs were able to increase DNA loading capacity and gene transfection efficiency and could be potentially applied in targeted gene delivery and therapy.

## Introduction

The success of gene therapy largely depends on the development of vectors or vehicles that can selectively and efficiently deliver genes to targeted cells with minimal toxicity. Generally, the gene delivery vectors can be divided into two categories: viral and non-viral. The former which uses replication-deficient viruses (such as retrovirus, adenovirus, adeno-associated virus and herpes simplex virus) has the advantage of high gene delivery efficiency, but is handicapped in clinical applications due to their immunogenicity, potential mutagenicity, low transgene size and high cost [1]. The non-viral vectors usually includ cationic liposomes, cationic polymers, synthetic peptides and naturally occurring compounds. Although the non-viral vectors have shown to be significantly less effective in vivo in comparison with the viral vectors, they are believed to attractive alternatives to viral vectors for their lack of specific immune response, versatility, ease of large-scale production and simplicity of usage [2]. Both gene therapies via viruses and non-viral vectors have potential to be treatment techniques particularly for gene-diseases, but the development of a safe and efficient gene delivery system is a long process which necessarily involves clinical trials [3], [4], [5].

Ultrasound targeted microbubble (MB) destruction (UTMD) is a physical gene transfection technique, known for being safe, effective, and non-invasive [6], [7], [8]. The MB, in addition to its well-known application as a contrast agent, has also been used as a drug/gene carrier, can be visualized and monitored in real time with assistance of ultrasound imaging. Cargo-loaded MBs can circulate easily within the vascular system until they reach a specific region of interest, and then they can be cavitated locally with high intensity focused ultrasound, causing site-specific delivery of the bioactive materials into cells through a process called sonoporation [9]. Excited by ultrasound, gas-filled MBs may oscillate drastically and eventually collapse via a process called inertial cavitation, releasing the energy necessary to induce transient cell membrane permeabilization [9]. Microstreaming and acoustic radiation force are also thought to contribute to gene uptakes [10], [11]. UTMD has been proposed as an innovative method for noninvasive gene delivery for different kinds of tissue.

Recently, the therapeutic effects of ultrasound-mediated gene delivery with MBs have been demonstrated both in cell culture [12], [13] and in vivo studies [14], [15], [16], [17]; however, the transfection efficiency was found to be low. One of the main reasons to low efficiency is the low DNA loading capacity of MBs. Simple blending of plasmid DNA with plain MBs, a method being most commonly performed, cannot upload enough DNA to MBs. Therefore it is difficult to achieve sufficient concentration of genetic material at the sonoporation site. Many strategies and formulations have been proposed to prepare DNA loading MBs. The methods include (1) preparing polymer MBs by using double-emulsion solvent evaporation method (w1/o/w2) and adding DNA to the inner water (w1) phase during the primary emulsification [18], (2) layer-by-layer (LBL) assembly technique to deposit multi-layers of cationic polymer on the MB shell to electrostatically bind DNA [19], (3) non-covalent coupling of RNA loaded cationic liposomes onto the MB surface via avidin-biotin interactions [20], (4) preparing cationic MBs by incorporating some cationic lipids such as DMTAP, DOTAP, DPTAP, DSTAP, DOTMA, or DDAB into the lipid MB shell to electrostatically bind DNA [12], [15], [21], [22]. Most experiments using the above-mentioned strategies have increased effectiveness of DNA loading. However, those methods were usually complicated and less-convenient in preparation or still not enough to promote intracellular delivery and trafficking to the nucleus.

The cationic polymer, polyethylenimine (PEI), has been widely used for gene transfection due to its strong DNA compaction capacity and intrinsic endosomolytic activity [23], [24], [25]. Also, the “proton-sponge” effect makes DNA/PEI complex escape from the phagolysosomes into the cytoplasm to minimize the enzymatic degradation in the lysosomes [25], [26]. During transfection, cDNA is released in the cytoplasm and is then trafficked uncoated by an inefficient mechanism into the nucleus. It has been demonstrated that polyethylenimine promote transgene delivery to the nucleus in mammalian cells [27].

In this in vitro study, we introduce a novel cationic lipid MB to enhance the DNA loading capacity of the MBs by coupling PEI onto the shell of the MBs. The concentration, size distributions, stability, zeta potentials and DNA loading capacity of the MBs were measured. The gene transfection efficiency and the cytotoxicity were also examined using breast cancer MCF-7 cells via the reporter plasmid pCMV-Luc, encoding the firefly luciferase gene.

## Materials and Methods

### Materials

1,2-distearoyl-sn-glycero-3-phosphocholine(DSPC) and 1,2-distearoyl-sn-glycero-3-phosphoethanolamine-N-[methoxy(polyethyleneglycol)-2000] (ammonium salt) (DSPE-PEG2000) were purchased from Avanti Polar Lipids (Alabaster, AL). Branched polyethylenimine (molecular weight  =  600 Dalton, PEI600), stearic acid and N, N'-carbonyldiimidazole (CDI) were purchased from Aladdin (Shanghai, China). FITC and Salmon sperm DNA were purchased from Sigma-Aldrich (St. Louis, MO). SYBR green was purchased from Invitrogen (USA). The human MCF-7 cancer cells were obtained from the American Type Culture Collection (ATCC). Cell Counting Kit-8 was purchased from Dojindo (Kumamoto Japan). Perfluoropropane (C_3_F_8_) was purchased from Huahe New-technology Development Company (Tianjin, China). Hoechst 33258 fluorescent dye for DNA labeling was obtained from Beyotime (Shanghai, China). All other chemicals were prepared with analytical grade reagents dissolved in 18.2 mΩ deionized water prepared by Milipore (Milli-Q Reference).

### Synthesis and characterization of stearic acid modied polyethylenimine 600 (Stearic-PEI600)

The Stearic-PEI600 was synthesized according to a previous report described by Wan et al[28]. In brief, 0.35 g (2.16 mmol) N, N'-carbonyldiimidazole (CDI) was dissolved in 10 ml anhydrous chloroform. 0.6 g (2.1 mmol) stearic acid was dissolved in dry chloroform (10 ml) and then added dropwise into upper CDI solution under magnetic stirring. The mixture was reacted at room temperature for 2 h under argon protection. The activated stearic acid was further added drop by drop to the dry branched PEI solution (0.7 g, 1.17 mmol, in 20 ml dry chloroform). The suspension was kept stirring at room temperature for further 24 h under argon protection. The resulting product was purified by precipitation in cold ether and collected by centrifuge at 3000 rpm for 10 min. The purified Stearic-PEI600 was further dried under high vacuum condition to remove trace amount of solvent.

### Preparation of plain and cationic MBs

The plain MBs were prepared by using mechanical agitation method reported in a previous publication [29]. As for the cationic MBs, the Stearic-PEI600 polymer was introduced. A lipid film, with molar percentages of 10% DSPE-PEG2000, N% Stearic-PEI600, and (90-N) % DSPC, was formed by removing the chloroform in the phospholipid solution under nitrogen flow, where N was variable. Residual chloroform was further eliminated under high vacuum for at least 2 h. A Liposome (3 mg/ml) suspension was produced by hydrating the dry lipid films with a given buffer consisting of 0.1 M Tris (pH 7.4, glycerol and propylene glycol (80∶10∶10 by volume). The suspension was then sonicated at 60°C for 5 min by a bath sonicator (40 kHz, 240 W). The resulting solution was sealed in a 3 ml serum vial (1 ml each) with a rubber cap and an aluminum seal. Finally, air in the vial was exchanged with C_3_F_8_ using a homemade apparatus. MBs were formed by shaking the vial with a vibrator for 45 s. The FITC-labeled Stearic-PEI600 was used to prepare fluorescent MBs and to show that the Stearic-PEI600 was incorporated into the shell of the MBs.

### Plasmid DNA and salmon sperm DNA preparation

The reporter plasmid pCMV-Luc, encoding the firefly luciferase gene under the control of the CMV promoter, was propagated in Escherichia coli TOP10, extracted and purified using plasmid extraction kit (NucleoBond® Xtra Midi EF) according to the manufacturer’s instructions. The concentration and purity were determined by measuring UV absorbance at 260/280 nm with a BioPhotometer (Eppendorf, Germany). Salmon sperm DNA was dispersed in deionized water by using a bath sonicator (40 kHz, 240 W), and the concentration was measured by UV spectrophotometry.

### Concentration and size distribution of MBs

After shaking the vial for 45 s using a vibrator, the obtained milky MB suspension was drawn into a 10-ml syringe and diluted to a final volume of 4 ml. MBs were washed with PBS three times in a bucket rotor centrifuge (ALLEGRAX-12R, Beckman Coulter, USA) at 400 g for 3 min at 4°C to remove excess free unincorporated lipids. The size distribution and concentration of MBs were measured using an Accusizer 780A (Particle Sizing System, Santa Barbara, USA). The freshly prepared MBs that were directly drawn from the vials right after shaking were also sampled.

### Zeta potential of the MBs

Zeta potential of the MBs was measured using a Zetasizer NANO ZS system (Malvern, UK). Before measurement, the MBs were washed with 10 mM sodium chloride solution or deionized water thrice as described above. Diluted MBs with a concentration of 1×10^8^ bubbles/ml were measured. All samples were measured three times.

### Characterization of plasmid DNA-loaded MBs

In order to determine the optimal ratio of MBs to plasmid DNA, the DNA-loaded MBs were resolved with 1% agarose gel stained with SYBR green (Invitrogen). In each experiment, different amounts of PEI600 MBs (1.0×10^6^, 2.0×10^6^, 4.0×10^6^, 8.0×10^6^, 1.2×10^7^, 1.6×10^7^, 2.0×10^7^ cationic MBs) were incubated with 0.36 µg DNA for 15 min to form the DNA/MB complexes. To maintain the same final volume, an appropriate amount of DNA loading buffer was added to each sample. Gel electrophoresis was carried out at 110 V for 20 min. Image of the gel was captured using a Gel Imaging System (Dolphin-Doc Plus).

To verify the coupling of the plasmid DNA and cationic MBs, Hoechst 33258 working solution was used to stain the DNA on the surface of MBs. The images were captured using a inverted fluorescence microscope (Leica DMI 3000B).

### DNA loading capacity of MBs

Salmon sperm DNA (Sigma-Aldrich) was used to measure the DNA loading capacity of cationic MBs as previous described [30]. Briefly, 2 mg/ml salmon sperm DNA was prepared by dissolving a proper amount of DNA in deionized water using a bath sonicator. An amount of 500 µl PEI600 MBs (resulting in 10^9^ MBs/mL) was slowly injected into 1 ml of DNA solution. The suspension was incubated by gentle rotation for 1 h to speed up the process of DNA adsorption onto MBs; the uncoupled free DNA was then removed by centrifugation and washing thrice in a centrifugal tube (400 g, 3 min). After the residual DNA loaded MBs were collected, the zeta potentials and size distributions were measured as mentioned.

The MBs were then destroyed in a bath sonicator heated about 65°C for several minutes until the suspension became transparent. The concentration of DNA in the suspension was measured using a BioPhotometer (Eppendorf, Germany), basing on 1 OD_260_ (i.e. a solution having an absorbance of one unit at 260 nm with a path length of 1 cm) corresponds to a concentration of 50 µg/ml for double-stranded DNA. All the measurements were made in triplicate. To reduce the discrepancies caused by the varied size distributions between samples, the DNA loading capacity of MBs were normalized by their total MB surface area. Assuming that MBs were all sphere shapes, and their total surface area of the measured sample could be estimated by the summation of surface area of all MBs in all channels sized by Accusizer 780A.

### Cell culture

Human breast cancer MCF-7 cells were employed to evaluate gene transfer and expression. The cells were maintained in Dulbecco's Modified Eagle Medium (DMEM), supplemented with 10% FBS and 1% penicillin-streptomycin solution and maintained in a humidified atmosphere containing 5% CO_2_ at 37 °C.

### Ultrasound-mediated gene transfer with MBs

Human breast cancer MCF-7 cells were seeded in 96-well plates and transfection experiments were performed when the cell confluence reached 70–80%. Plain MBs/DNA and PEI600-MB/DNA complexes were prepared by premixing and incubating 10 µl DNA with 10 µl plain MBs or 5 mol% PEI600 MBs (20 µl total mixture volume in 0.9% NaCl) for 30 min at room temperature (25 °C) to allow for spontaneous binding of the anionic DNA and MBs. Then the complexes were added into each well, and the final concentrations of the DNA and MBs were maintained at 2 µg/ml and 10^7^/ml, respectively. 10 µl DNA and 10 µl MBs without premix were added to each well of a control group successively. After the 96-wells plate was sealed firmly and turned upside down for 15 min to allow the DNA/MBs complexes to float and adhere to the cell monolayer, ultrasound exposure was performed.

The ultrasound system used in this experiment includes an arbitrary waveform generator (model AFG3102, Tektronix, USA), an RF power amplifier (model AR150A100B, AR, USA), and a weakly focused transducer (Valpey Fisher, MA, USA) whose center frequency is 1.2 MHz, focal length is 5.0 cm, diameter is 5.1 cm, and f-number is close to unity. A 120-cycle sinusoidal tone-burst with 1 kHz pulse repetition frequency and an acoustic pressure amplitude of 0.6 MPa, which was measured in situ using a needle hydrophone of resolution 1 mm, was used to treat each sample for 15 s. Then the treated plates were incubated for 6 h in a humidified atmosphere containing 5% CO_2_ at 37°C. After that, the medium was replaced with fresh DMEM with 10% FBS and 1% antibiotics. 24 h after transfection, 100 µl DMEM containing D-luciferin (150 µg/mL final concentration) was added to each well and the luciferase expression of these MCF-7 cells was evaluated using a Xenogen IVIS-100 system (Caliper Life Sciences).

### Cell viability assay

The cytotoxicities of PEI600 MBs with or without ultrasound exposure were measured and compared with Plain MBs. 24 h after transfection, the cell viability was evaluated using Cell Counting Kit-8 (Dojindo, Kumamoto, Japan) according to the manufacturer’s protocol. The absorbance at 450 nm was measured by using a multimode plate reader (Synergy 4, BioTek).

### Statistical analysis

Statistical analysis was performed using the two-tailed t-Test method assuming unequal variances. A p value of <0.05 was considered to be statistically significant.

## Results

### PEI modification and PEI600 MBs

**Figure 1:**
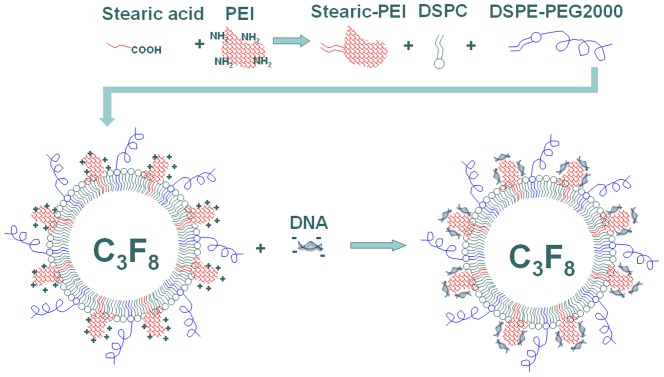
Illustration of the PEI modification by stearic acid for preparing of cationic microbubbles (MBs) to load DNA. The stearic acid modified polyethylenimine 600 (Stearic-PEI) polymer was synthesized. The resulting Stearic-PEI, combined with DSPC and DSPE-PEG2k, was used to fabricate the cationic MBs. The PEI endows the cationic MBs with more amino groups to couple DNA than plain MBs.

**Figure 2:**
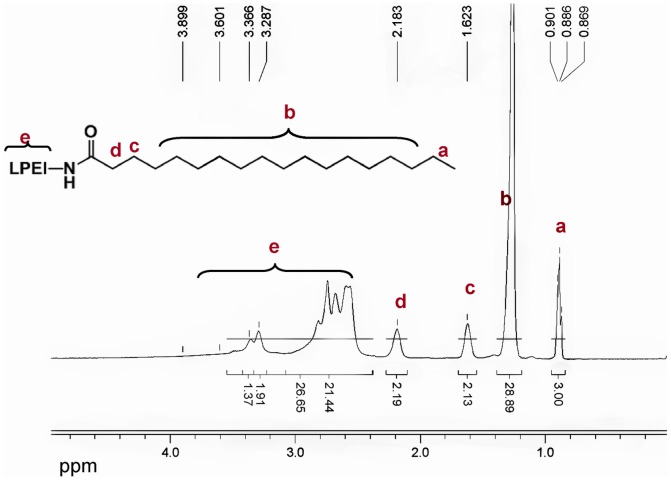
^1^H NMR spectrum of Stearic-PEI. (CDCl_3_), δ (ppm) 0.86–0.89 (t, -CH_2_CH_2_(CH_2_)_15_C**H_3_**), 1.25 (br, -CH_2_CH_2_(C**H_2_**)_15_CH_3_), 1.62 (br, -CH_2_C**H_2_**(CH_2_)_9_CH_3_), 2.18 (br, -C**H_2_**CH_2_(CH_2_)_9_CH_3_), 2.39–3.3 (m, -CH_2_C**H_2_**NH-, -CH_2_C**H_2_**N-, -CH_2_C**H_2_**NHCO-, -C**H_2_**CH_2_NHCO-).

As schematically presented in Fig. 1, branched PEI600 (MW  =  600 Da) was modified with stearic acid via acylation reaction at a molar ratio of 1∶2. Hydrophobic stearic chains were introduced onto the branched PEI by CDI. The molecular structure of the product was confirmed by ^1^H NMR in CDCl_3_. ^1^H NMR analysis indicates that 14% amino-groups of the PEI600 were acylated (Fig. 2). Each PEI600 molecule is connected with nearly two stearic chains and has a very similar structure with the phospholipids used to fabricate the shell of MBs [31].

**Figure 3:**
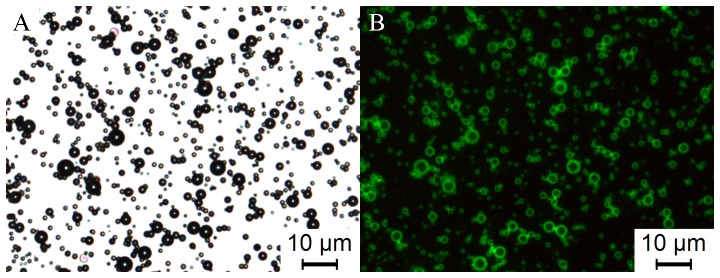
The bright field and fluorescence images of FITC-labelled Stearic-PEI600 cationic MBs. (A) Bright field image and (B) fluorescent image of MBs containing 5% Stearic-PEI600. Scale bars: 10 µm.

As shown in Fig. 1, the modified PEI was incorporated into the shell of the MBs by hydrophobic-hydrophilic interactions. Fluorescence images confirm that the Stearic-PEI600 was partially embedded into the shell of the MBs (Fig. 3). The bright field image shows bright gas cores of the MBs surround by dark circular rings (Fig. 3A). Some of the MBs seem to be a bit fuzzy because they were out of the focus. The fluorescence image shows the opposite pattern with dark cores and bright green shells (Fig. 3B). They demonstrate the FITC-labeled Stearic-PEI600 cationic MBs have been established.

### Concentration, size distribution of PEI600 MBs

**Figure 4:**
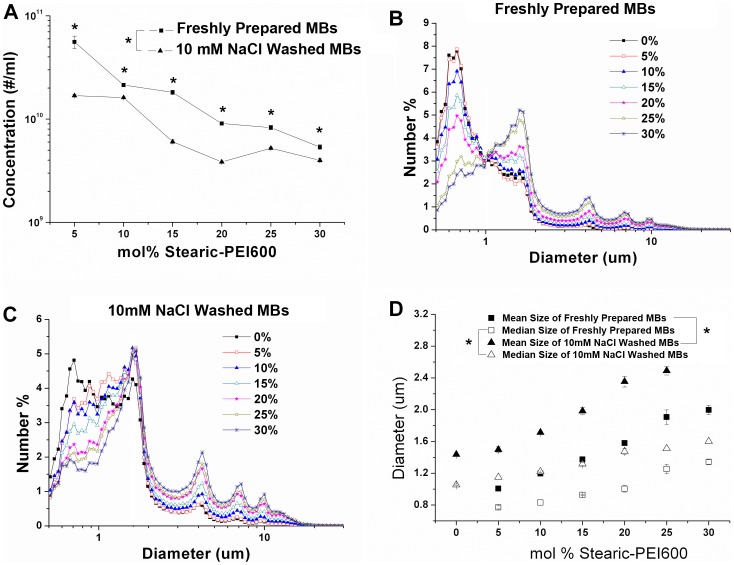
Concentration and size distribution of fresh prepared and washed MBs. (A) Concentrations of freshly prepared and washed MBs with various amounts of Stearic-PEI600. (B) Size distribution of freshly prepared MBs with various amounts of Stearic-PEI600. (C) Size distribution of 10 mM NaCl washed MBs with various amounts of Stearic-PEI600. (D) Mean and median size of fresh prepared and 10 mM NaCl washed versus the Stearic-PEI600 fraction. The “freshly prepared” MBs were taken straight from the vial within 1 h of formation. The “washed” MBs were obtained by three cycles of flotation-centrifugation with infranatant exchange to remove the submicrometer bubbles.

Fig. 4A shows that the concentration and size distribution of the resulting cationic MBs. The concentrations, the number of MBs per ml, of both freshly prepared MBs without wash and that washed with 10 mM NaCl, gradually decreased with the increase of Stearic-PEI600 in cationic MB formulations. Compared with the concentration of 5 mol% PEI600 MBs, the concentration of 30 mol% PEI600 MBs had a nearly 90% decrease in MB concentration (P< 0.01) for freshly prepared MBs and a 75% decrease for NaCl-washed MBs respectively. The size distributions of the freshly prepared and NaCl-washed MBs were presented in Fig. 4B and Fig. 4C. The submicron bubbles (size <1 µm) dominated in the freshly prepared MBs when the amount of Stearic-PEI600 used for the cationic MB fabrication was less than 20 mol%. The size distributions of NaCl-washed MBs showed a similar trend (Fig. 4C). The ratios of the submicron bubbles were reduced from 68.3% to 27.7% for fleshly prepared MBs and from 45.9% to 20.8% for NaCL washed MBs with the increased amount of Stearic-PEI600 from 5% to 30%, which were in agreement with the previously reported results [30]. The mean and median sizes of freshly prepared MBs and washed MBs are shown in Fig. 4D and which show a gradually increase in the mean and median diameters of MBs with the increased amount of Stearic-PEI600 from 0% to 30%. Moreover, NaCl-washed MBs also have larger mean and median diameters than freshly prepared MBs (P<0.05).

### Zeta potential of the PEI600 MBs

**Figure 5:**
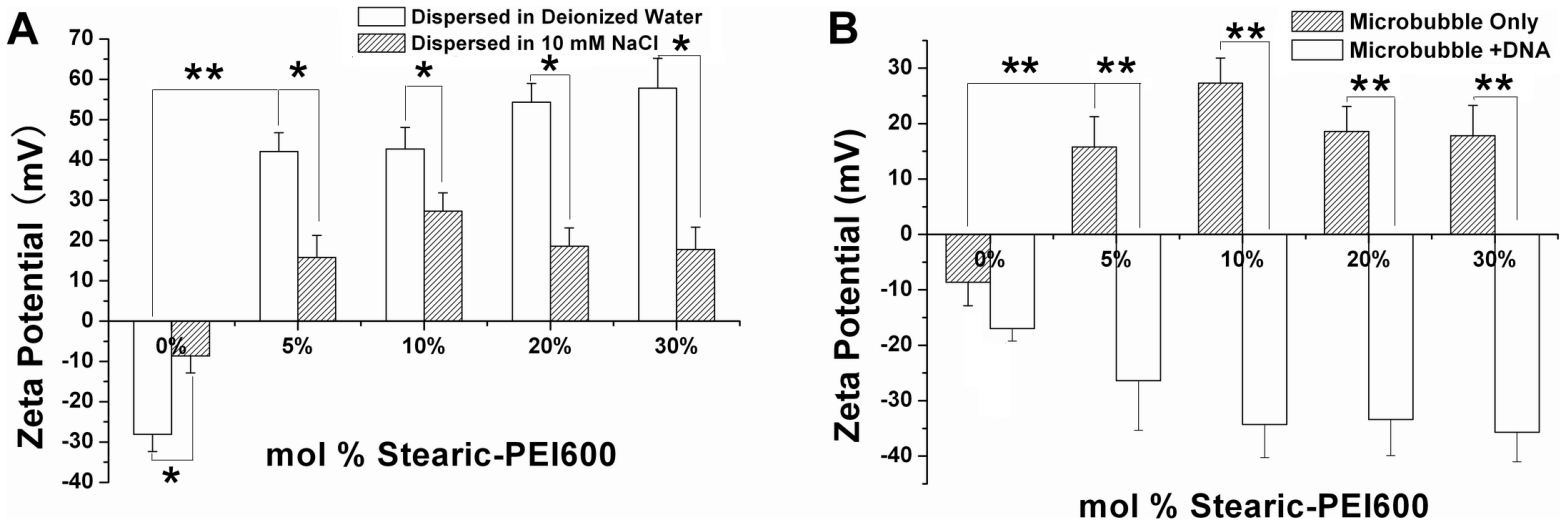
The zeta potential of PEI600 MBs. (A) Zeta potential of MBs dispersed in deionized water and 10 mM NaCl. (B) Zeta potential of MBs binding with and without DNA.

Zeta potential of the cationic MBs is significant for DNA binding through the electrostatic interactions. The detection of laser Doppler anemometry showed that Stearic-PEI600 had a significant effect on *ζ-*potential of MBs. Plain MBs without Stearic-PEI600 (0%) had a *ζ-*potential of –28.1±4.3 mV in deionized water due to the presence of negatively charged phosphate groups in the DSPE-PEG2000. Addition of 5%, 10%, 20% or 30% Stearic-PEI600 during MB preparation resulted in a dramatic increase of the MB surface ζ-potentials from negative to positive; they reached 42.1±4.65 mV, 42.70±5.34 mV, 54.30±4.70 mV, 57.80±7.41 mV for MBs in deionized water, respectively. It was noted that the *ζ-*potentials of all kinds of MBs in 10 mM NaCl decreased in comparison with those of MBs dispersed in deionized water (Fig. 5A). MBs without Stearic-PEI600 had a negative *ζ-*potential of –8.61±4.26 mV when dispersed in 10 mM NaCl, with a 19.49 mV decrease of the magnitude of MB surface potential (P<0.01), compared with sample without Stearic-PEI600. Similarly, there were 26.3 mV (P<0.01), 15.4 mV (P<0.01), 35.7 mV (P<0.01) and 40 mV (P<0.01) decreases of the magnitude of *ζ-*potential for 5%, 10%, 20% and 30% PEI600 MBs, respectively. Fig. 5B showed the *ζ-*potentials of MBs coated with the Stearic-PEI600 and that saturated by excess salmon sperm DNA. Interestingly, the addition of DNA into cationic MB suspension in 10 mM NaCl dramatically reversed the *ζ-*potentials of MBs from positive to negative (Fig. 5B). when saturated by excess salmon sperm DNA, the surface potentials of 5%, 10%, 20% or 30% Stearic-PEI600 cationic MBs were –26.40±8.93 mV, –34.30±5.95 mV, –33.40±6.49 mV and –35.70±5.35 mV, achieving decreases of 42.20 mV (P<0.01), 61.60 mV (P<0.01), 52.00 mV (P<0.01) and 53.50 mV (P<0.01) respectively. However, there was no significant decrease of the magnitude of surface potentials for plain MBs without Stearic-PEI600 (P > 0.05).

### Plasmid DNA binding onto PEI600 MBs

**Figure 6:**
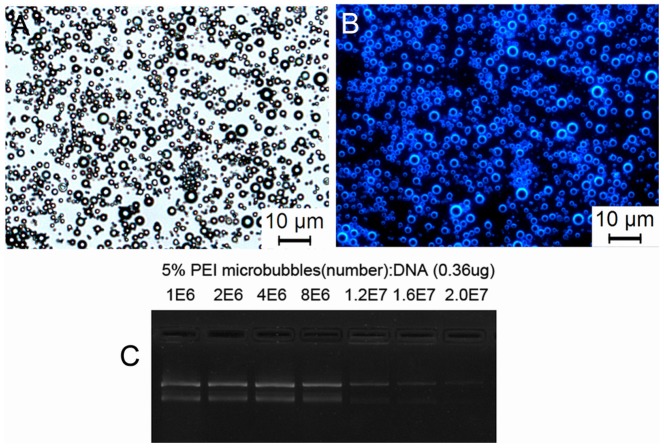
Binding of plasmid DNA onto PEI600 MBs. (A) The bright field image and (B) the corresponding fluorescent image of plasmid DNA loaded MBs. (C) Agarose gel electrophoresis of 0.36 µg plasmid DNA mixed with different number of MBs with 5% Stearic-PEI600.

In order to visually examine whether the PEI600 MBs could bind with plasmid DNA, Hoechst 33258 was used to stain plasmid DNA on the cationic MBs. The bright field image showed the shape of DNA-MB complexes were not affected by DNA-binding (Fig. 6A). The blue surface of the DNA-binding MBs observed under fluorescent microscope (Fig. 6B), which indicated successful adhesion of the negatively charged plasmids to the surface of cationic MBs.

Agarose gel electrophoresis of cationic MB/DNA complexes at various MB/DNA ratios was presented in Fig. 6C. Reduced or no migration of the DNA into the gel at ≥ 2.0×10^7^ MB/µg DNA ratio indicated the complexes formation. 0.36 µg DNA was completely captured by 2.0×10^7 ^MBs, responding to the ratio 5.5×10^7 ^MBs coupled 1 µg plasmid DNA.

### DNA loading capacity of PEI600 MBs

**Figure 7:**
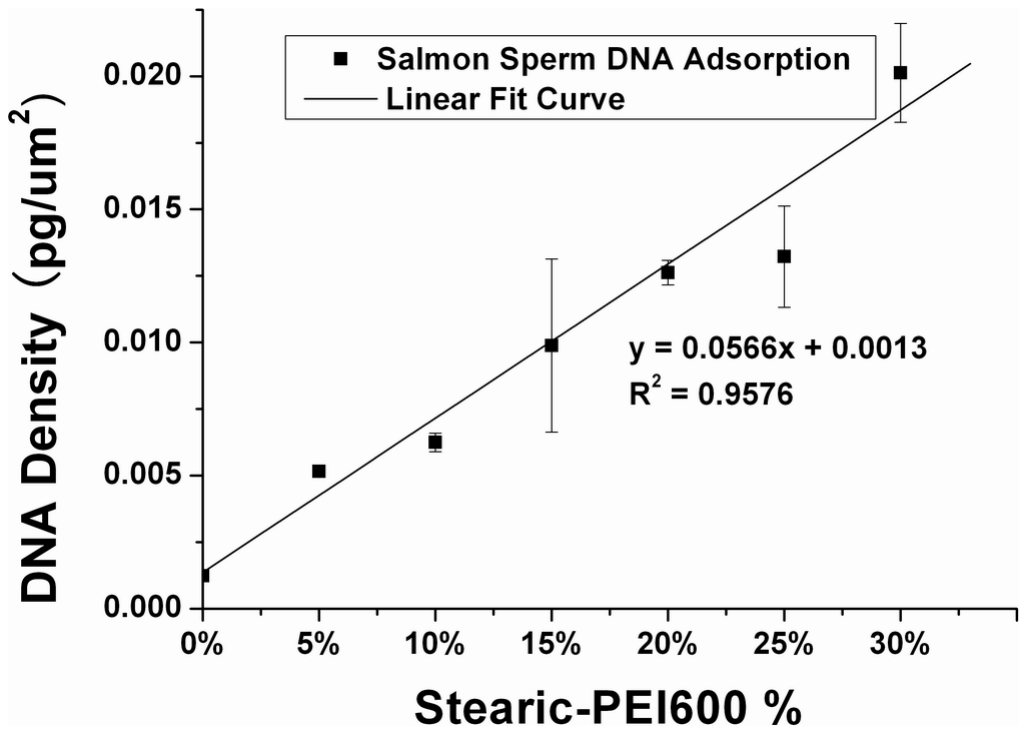
DNA loading capacity vs Stearic-PEI600 (%). The relationship between DNA loading capacity (y) and Stearic-PEI600% (x) can be fitted to a linear rewlation : y = 0.0566x+0.0013 with R^2^  =  0.9576. Salmon sperm DNA surface concentrations were obtained by analyzing the 260 nm absorbance and corresponding total surface area of sized MBs.

The DNA-loading capacity was quantitatively determined through calculating the DNA density on the MBs according to the previous publication [30], [32]. From Fig. 7 we can see that the DNA density on the MBs increased nearly linearly from (5±0.2)×10^−3^ pg/µm^2^ to (20±1.8) ×10^−3^ pg/µm^2^ when Stearic-PEI600 increased from 5 mol% Stearic-PEI600 to 30 mol%. Since the average molecular weight of one DNA base pair is about 670 Daltons, a 2 µm 5% PEI600 MB would couple 0.0628 pg DNA, corresponding to about 6×10^7^ base pairs. 10^7^ MBs would bind 0.68 µg DNA, which was in the same order of magnitude with the estimated data from gel electrophoresis.

### Ultrasound-mediated transfection in cell culture

**Figure 8:**
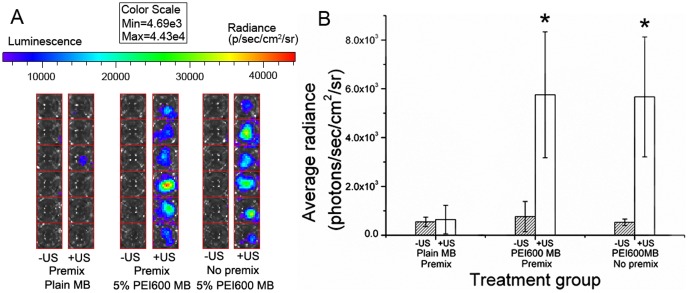
DNA transfection in MCF-7 cells. (A) Optical images of luciferase gene expression. (B) Bar graph shows the luciferase activity in MCF-7 cells after ultrasound-mediated transfection of pCMV-Luc with complex of DNA/Plain MBs, complex of DNA/5% PEI600 MBs by premix DNA and MB for 15minutes, and with 5% PEI600 MB and DNA without premixing and added separately. pCMV-Luc activities were evaluated 24 h after gene transfection.

Transfection of MCF-7 cells using DNA/5% PEI600 MBs complex plus ultrasound exposure yielded 5.76±2.58×10^3^ p/s/cm^2^/sr average radiance intensity, was 8.97- and 7.53-fold higher than those treated by plain MBs with ultrasound exposure (6.41±5.82×10^2^ p/s/cm^2^/sr, P<0.01) and by PEI600 MBs without ultrasound (7.65±6.18×10^2^, p/s/cm^2^/sr, P<0.01), respectively. And the cells treated by adding PEI600 MBs and DNA (without premix) with ultrasound yielded an average radiance 5.67±2.46×10^3^ p/s/cm^2^/sr, is was 8.84-fold higher than treated by plain MBs and ultrasound exposure. The samples treated by ultrasound exposure with or without premixing PEI600 MBs and DNA made no difference in the plasmid DNA transfection efficiency (Fig. 8).

### Cytotoxicities

**Figure 9:**
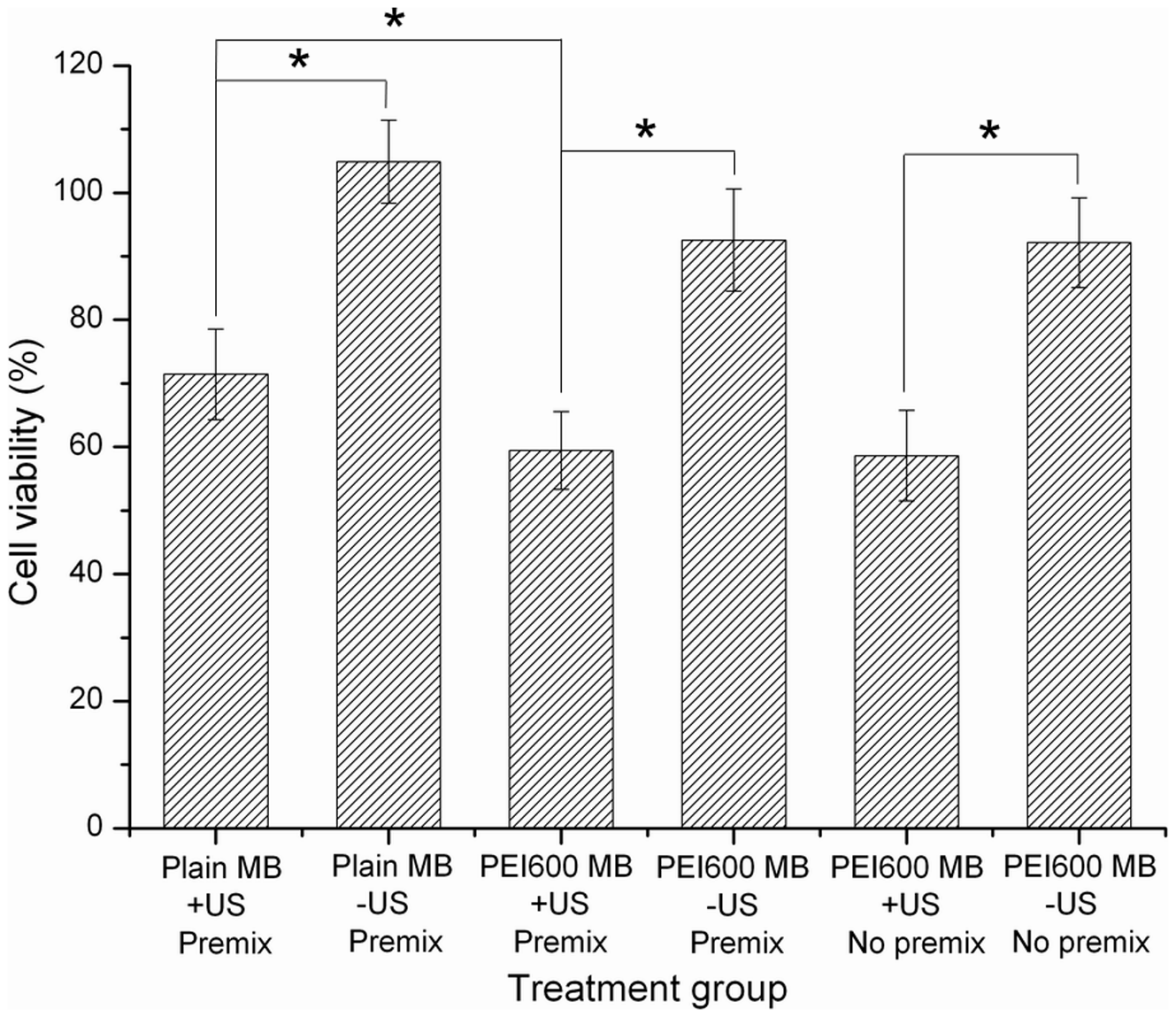
Cell cytotoxicity assay. The cell viability was measured by using the CCK-8 assay after 24 h transfection of pCMV-Luc with with complex of DNA/Plain MBs, complex of DNA/5% PEI600 MBs by premix DNA and MB for 15minutes, and with 5% PEI600 MB and DNA without premixing and added respectively, with or without ultrasound exposure.

The cell viability was measured by using CCK-8 assay. The cells treated with DNA/PEI600-MBs and DNA/plain MBs complexes under ultrasound exposure showed 59.5±6.1% and 71.4±7.1% cell viability, respectively. No matter with and without ultrasound exposure, the cytotoxicitiy of PEI600 MB was slightly larger than plain MBs (P<0.01), and ultrasound exposure showed a strong toxicity in every groups (P<0.001). There were not significant differences in cell viability whether the DNA and PEI600MB were premixed or not (Fig. 9). Thus, it was indicated that the cytotoxicity may be mainly caused by the MB cavition induced by ultrasound exposure, however, the PEI600 MBs showed higher cytotoxicity than plain MBs.

## Discussion

Recently, most MB-mediated gene delivery experiments were performed by co-injection of MBs and DNA vectors. In those studies, circulating MBs were insonified to increase local vascular permeability, allowing DNA vectors to passively extravasate into tissue. However, circulating genetic agents may also extravasate into fenestrated organs (such as the lung, liver, and spleen), resulting in off-target effects [33]. Strategies such as DNA-loaded polymer MBs using double-emulsion solvent evaporation method (w_1_/o/w_2_), layer-by-layer (LBL) assembly technique and DNA-loaded liposome-MB complexes have been proposed to overcome this problem [30], [24], [13]. Another strategy is to incorporate directly cationic polymer such as DSTAP into the MB shell. The former can increase effectiveness of DNA loading, but are often complicated and less-convenient during preparation. The latter also has some drawbacks. For example, the DNA loading capacity of the MBs would easily be saturated at a low concentration level due to the limited surface area of MBs. Contrarily, in our study, DNA vectors were attached to the surfaces of the PEI600-modified cationic MBs, forming MB-DNA hybrid vectors. In consideration of its relatively low cytotoxicity compared with that of mostly used PEI 25000 [31], PEI600 was selected to fabricate the cationic MBs. By attaching DNA onto the MB surface, their release can be mediated by acoustic cavitation around the ultrasound focal zone, thus providing more specific control in tissue-targeting applications [34]. Furthermore, attachment of DNA to the MB surface has potentially provided some protection, preventing rapid clearance of DNA [21], [35]. Several studies have demonstrated that DNA molecules loaded onto MBs improves their intracellular uptake in vitro [36], [37] and deposition into target tissue in vivo [38]. Wang et al. showed that plasmid-binding cationic MBs had enhanced ultrasound-mediated gene delivery efficiency relative to neutral MBs in both cell culture and mouse hind limb tumors [21], [39].

In the current study, we found that the MB size, concentration and size distributions were significantly impacted by the molar amount of Stearic-PEI600 in preparing the cationic MBs. Our results were in agreement with the previous reports. Borden et al. have reported the lateral phase separation in lipid-coated MBs [40]. The ordered domains in the shell of MBs are composed primarily of DSPC, while the disordered interdomain regions are mainly lipopolymer. Thus, increasing the cationic polymer concentration would generally result in disorder domains increase and condensed domains reduction. These disorder domains would increase the mass transfer, so that stability was weaken. In another study, Feshitan et al. found that higher concentration MBs, in general, tended to be more stable, regardless of MB size [41].

Surface charge of the cationic MBs indicated a significant change in the surface of MBs after addition of the cationic polymer Stearic-PEI600 (Fig. 5A). This is mainly because that the ionic strength of the polyelectrolyte such as PEI is determined not only by the concentration of the polyelectrolyte itself, but also the concentration of small molecule electrolytes such as NaCl. The protonated amine group of the PEI is shielded to some extent by the counter-ions in the NaCl solution. It was demonstrated that the zeta potential was sensitive to both pH and the presence of ionic character of the suspending medium. Nomikou et al. measured a lipid-shelled MB with a 8% molar ratio of DSTAP and described a zeta potential of approximately 4–5 mV in Opti-DMEM [42], while Borden et al. described a MB zeta potential of approximately 33 mV with a 20% molar ratio of DSTAP in 10 mM NaCl, which was closed to our results [30].

It is notable that a significant improvement of DNA loading capacity was achieved in our new designed cationic MBs. The improvement of DNA loading capacity may contribute to the abundant amino group in PEI molecules. Each PEI600 molecule has approximately 14 nitrogen atoms, and at most 12 residual nitrogen atoms of which could be protonated to adsorb DNA after being modified by two stearic acid molecules. So, ideally, the maximum DNA loading capacity should be ten folds more than that of the traditional cationic MBs which consist of the same molar ratio of polymers such as DSTAP. Herein, DNA loading capacity of PEI600 MB we measured is about five times higher than that of the reported DSTAP MBs, which may be because of the steric hindrance. Borden et al. applied a layer-by-layer (LBL) assembly technique to adsorb multiple layers of DNA and poly-L-lysine (PLL) onto lipid-coated MBs [30]. The DNA loading capacity was enhanced by over 10-fold by using five paired layers. Nevertheless, LBL assembly technique is somewhat complex as the fragility of the MBs.

It has been proved that DNA could be effectively protected from degradation by coupling them to the cationic MBs via electrostatic interactions [35], [43]. In recent studies, the gene transfer by using UTMD with injecting MBs and DNA/PEI complex simultaneously have shown that UTMD could improve the gene transfection efficiency of DNA/PEI complex in vitro and in vivo [17], [44]. Sirsi et al. also reported that DNA/PEI-MBs could transfect tumor tissue in a site-specific manner by virtue of ultrasound. In their study, the branched polyethylenimine (PEI) was first modified with polyethylene glycol and hydrosulfide group, and then covalently attached to the maleimide groups on the shell of the MBs that contain a functionalized PEG lipid. Thus, DNA could be adsorbed by the PEI coated MBs.

In this study, the DNA transfection was carried out under the following conditions: frequency  =  1.2 MHz, the acoustic pressure amplitude  =  0.6 MPa, a 120-cycle sinusoidal tone-burst with 1 kHz pulse repetition frequency, MB concentration  =  1×10^7 ^MBs/ml, DNA concentration  =  2 µg/ml. In fact, the UTMD for DNA transfection in vitro and in vivo has been studied in similar conditions. It has been demonstrated that the DNA transfection efficiency depends on multiple factors, such as experimental systems, MB concentration, DNA concentration, acoustic intensity and pulse sequence, etc. [45], [46], [47]. Interestingly, as for the UTMD strategy for DNA transfection, it seems that the cell viability and transfection efficiency are contradictory. For example, transfection efficiency increased approximately linearly with MB concentration, but cell viability inversely correlated with MB concentration. The relationship between acoustic intensity and transfection efficiency was highly nonlinear. Clearly, the goal of this technology is to obtain high transfection efficiency and low cell death. However, the acoustic intensity at site, MB concentration and pulsing sequence may need to be carefully adjusted to obtain an optimal conditions, considering the tradeoffs between the transfection efficiency and cell viability [12].

## Conclusions

We have developed a cationic MB by modifying PEI with stearic acid and coating it onto the shell of MBs. The addition of the modified cationic polymer may affect the yield and size distribution of the MBs by forming a disorder domain in the shell of the MBs. Also, the buffer used to dilute and wash the MBs will affect both the size distribution and the zeta potential of the MBs due to the shielding effect of the small molecular electrolyte. The plasmid DNA can be effectively coupled onto the surface of the cationic MBs. Additionally, our results demonstrated an increasing DNA-loading capacity with increase of the Stearic-PEI600. DNA transfection of MCF-7 cells using the Stearic-PEI600 MBs and ultrasound is significantly higher than that of treatment with plain MBs with ultrasound, PEI600 MBs without ultrasound respectively. And premix the PEI600 MBs with DNA or not doesn’t affect the transfection efficiency significantly in vitro. Our study may have laid down a foundation for image-guided gene therapy which we plan to explore in future.
